# Patient-Specific Quality Assurance Using a 3D-Printed Chest Phantom for Intraoperative Radiotherapy in Breast Cancer

**DOI:** 10.3389/fonc.2021.629927

**Published:** 2021-03-15

**Authors:** Yeonho Choi, Ik Jae Lee, Kwangwoo Park, Kyung Ran Park, Yeona Cho, Jun Won Kim, Ho Lee

**Affiliations:** ^1^ Department of Radiation Oncology, Gangnam Severance Hospital, Yonsei University College of Medicine, Seoul, South Korea; ^2^ Department of Radiation Oncology, Yonsei Cancer Center, Yonsei University College of Medicine, Seoul, South Korea; ^3^ Department of Radiation Oncology, Kosin University College of Medicine, Busan, South Korea

**Keywords:** intraoperative radiation therapy (IORT), patient-specific quality assurance, 3D printing, INTRABEAM™, breast cancer

## Abstract

This study aims to confirm the usefulness of patient-specific quality assurance (PSQA) using three-dimensional (3D)-printed phantoms in ensuring the stability of IORT and the precision of the treatment administered. In this study, five patient-specific chest phantoms were fabricated using a 3D printer such that they were dosimetrically equivalent to the chests of actual patients in terms of organ density and shape around the given target, where a spherical applicator was inserted for breast IORT treatment *via* the INTRABEAM™ system. Models of lungs and soft tissue were fabricated by applying infill ratios corresponding to the mean Hounsfield unit (HU) values calculated from CT scans of the patients. The two models were then assembled into one. A 3D-printed water-equivalent phantom was also fabricated to verify the vendor-provided depth dose curve. Pieces of an EBT3 film were inserted into the 3D-printed customized phantoms to measure the doses. A 10 Gy prescription dose based on the surface of the spherical applicator was delivered and measured through EBT3 films parallel and perpendicular to the axis of the beam. The shapes of the phantoms, CT values, and absorbed doses were compared between the expected and printed ones. The morphological agreement among the five patient-specific 3D chest phantoms was assessed. The mean differences in terms of HU between the patients and the phantoms was 2.2 HU for soft tissue and −26.2 HU for the lungs. The dose irradiated on the surface of the spherical applicator yielded a percent error of −2.16% ± 3.91% between the measured and prescribed doses. In a depth dose comparison using a 3D-printed water phantom, the uncertainty in the measurements based on the EBT3 film decreased as the depth increased beyond 5 mm, and a good agreement in terms of the absolute dose was noted between the EBT3 film and the vendor data. These results demonstrate the applicability of the 3D-printed chest phantom for PSQA in breast IORT. This enhanced precision offers new opportunities for advancements in IORT.

## Introduction

Intraoperative radiation therapy (IORT) is a treatment modality that entails accelerated partial breast irradiation for early-stage breast cancer patients (1, 2). IORT generally refers to the direct delivery of a single-fraction dose of highly localized radiation to the periphery of the lumpectomy bed during surgery (3). Its major advantage is that it offers the direct visualization of the tumor bed without incurring the risk of a marginal miss. This helps minimize damage to the healthy tissue by reducing the volume and dose of radiation to the normal surrounding tissue (4). Furthermore, compared with conventional whole-breast irradiation (WBI) over 5 to 5.5 weeks followed by tumor-bed boost or hypofractionated WBI over 3 weeks with a boost, IORT is completed in one day, and intraoperative irradiation allows for the immediate treatment of the surgical bed in 30 min to avoid a delay between surgery and external beam radiotherapy. This is convenient for the patient and helps reduce cost. Treating a smaller volume of normal tissue instead of performing WBI enables the reduction of potential lung and cardiac toxicities arising from radiation treatment and enhances tumor control (5–7).

IORT requires specialized radiotherapy equipment. In this regard, INTRABEAM™ (Carl Zeiss Surgical GmbH, Oberkochen Germany) uses a low-energy (50 kV) X-ray generator to provide partial-breast irradiation (8). This system uses spherical applicators to deliver a uniform dose on the inner surface of the breast lumpectomy cavity and irradiates high-dose (10–20 Gy) beams at once. Therefore, it is essential to ensure safe and accurate delivery through a patient-specific quality assurance (PSQA) process that verifies that the treatment device is physically capable of delivering the expected dose distribution prior to patient treatment. Because the radiation dose cannot be measured directly in the patient, it is common to create phantoms that mimic human radiation characteristics. Patient-specific dose measurements are often performed using radiation therapy phantoms combined with various dosimeters. These phantoms are made of homogeneous materials that simulate representative organs. However, commercially available phantoms for IORT are not supported for clinical use. Moreover, because most IORT clinics do not have a treatment planning system (TPS), a thorough understanding of the dose distribution of IORT is essential for safe, effective, and efficient treatment delivery. Special attention needs to be paid to all aspects of the treatment for each patient. However, it is difficult to perform accurate predictions regarding IORT because the volume of the tumors removed may vary depending on the surgery outcome. Thus, pretreatment planning and PSQA are limited (9, 10).

There have been attempts to manufacture customized objects incorporating 3D printing technology into various applications of radiation therapy (11–17). In this manner, to overcome the limitations of IORT’s PSQA, we ensure the stability of IORT by creating a PSQA phantom via 3D printing. This study investigates the feasibility of verifying IORT dosimetry using a 3D-printed water-equivalent phantom as well as patient-specific 3D chest phantoms fabricated by simulating the actual structure of the body of the patient around the target. We explore the effectiveness of the 3D-printed phantoms fabricated by considering the infill ratio corresponding to the average HU value assigned to the patient’s CT. We also examine the qualification and quantification of the depth dose and the dose administered on the surface of the applicator to ensure that the delivered dose matches the expected dose.

## Materials and Methods

### INTRABEAM™ System

The INTRABEAM™ system consists of a miniaturized accelerator (XRS) that accelerates electrons through a 10-cm drift tube, with a maximum voltage of 50 kV, onto a gold target where low-energy photons are produced and then emitted isotropically. An internal radiation monitor is used to detect the X-ray photons emitted in the direction of the cathode and record the dose output in real time. The miniaturized accelerator is inserted into the arm of the INTRABEAM carrier, which can be moved smoothly to any position in the operating room owing to the integral casters located at its base. Weight compensation and six axes provide sufficient freedom to place the miniaturized accelerator in any position in 3D space for access to the targeted area. Electromagnetic brakes hold the miniaturized accelerator in the exact set position during treatment. The operator can know the dose being delivered at any time throughout the treatment through the online dose monitoring data displayed on the treatment screen of the control terminal on the INTRABEAM cart. Spherical applicators are used for the intracavitary or intraoperative delivery of radiation to the tumor bed, e.g., during breast-conserving surgery. The applicator fills the cavity created by the excision of the tumor. The tissue on the tumor bed adheres to the applicator *via* surface tension. The probe tip is centered within the applicator and, therefore, at the tumor cavity. The INTRABEAM spherical applicators, which are reusable and sterilizable, are available in 5 mm increments, with diameters ranging from 15 mm to 50 mm. In this study, applicators with a diameter of 35 mm were used.

### Absolute Dosimetry Using Zeiss Water Phantom

Zeiss supplies a special water phantom consisting of a 3D translational stage for precise source positioning and a PTW 34013 soft X-ray ionization chamber (18). To calculate the dosage rate in water for the XRS using this phantom, Eq. 1 is suggested in the user manual (19):

(1) $$DR_{w}^{Zeiss}=M_{raw}P_{TP}N_{k}K_{Q}K_{K_{Q}\to D_{W}},$$

where *N_k_* is the ion chamber calibration factor (Gy/nC), *M_raw_* is the ionization charge (C) collected in 60 s for a chamber located at depth z in water, and *P_TP_* is the correction factor for room temperature (T) and pressure (P) at the time of dose measurement. The beam quality correction factor, *K_Q_*, was set to unity (*K_Q_* = 1) based on the fact that the T30 spectrum used as a reference X-ray beam, with E_eff_ =16.4 keV and HVL=0.43 mm AI, best matched the INTRABEAM spectrum (18). $K_{K_{Q}\to D_{W}}$ was the chamber conversion factor that converted air kerma measurements into doses in water for the chamber in the T30 spectrum ($K_{K_{Q}\to D_{W}}$ = 1.045). The manufacturer provided the calibration depth dose curve, where the measured $DR_{w}^{Zeiss}$ was plotted as a function of depth z.

### Fabrication of 3D-Printed Customized Phantom

**Figure 1:**
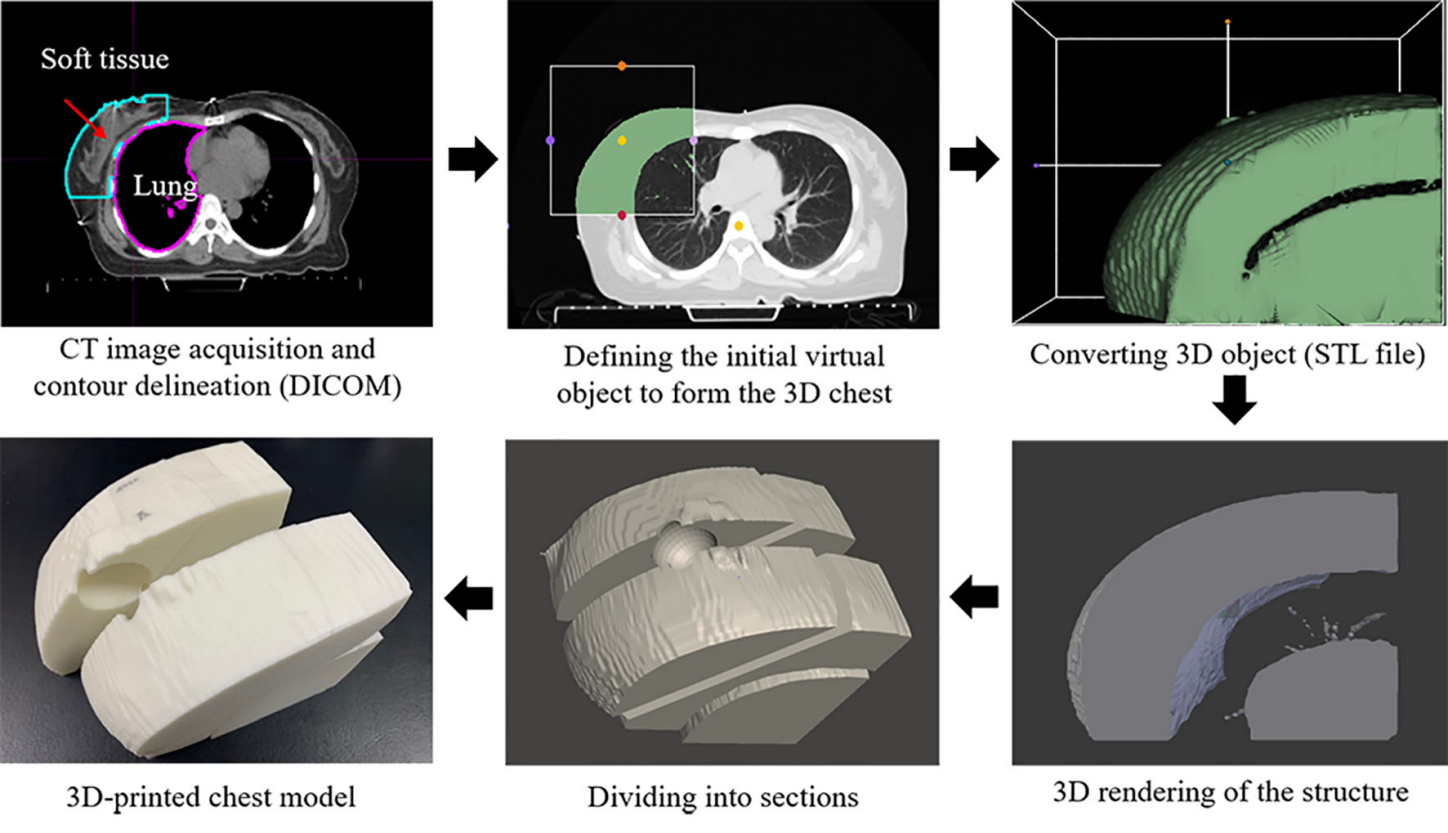
Schematics of creating 3D-printed chest phantom.

**Figure 1** illustrates the schematic design of the customized chest phantom fabricated by the 3D printer. CT image data for each patient, in the digital imaging and communications in medicine file format, were used to create a virtual 3D-printed chest phantom. A CT slice was collected every 3 mm and reconstructed as a 1-mm-spaced slice. Modifications to the CT images were performed using the MIM Maestro software (version 6.1, MIM Software Inc., USA). The growth in the seed region, followed by a manually adjusted 2D brush, was used to select the region of interest (ROI) that covered one side of the lung close to the tumor and the ROI that corresponded to the soft tissue including the breast. The mean HU values of voxels in each ROI were calculated.

3D Slicer was used to define the initial virtual object to form the 3D chest phantom based on the patient’s CT data. The virtual object with surface information in terms of triangular meshes was stored in the stereolithography (STL) file format, and it could be read by the 3D printer software. Using Blender, the converted raw 3D object was refined by disregarding any defective meshes using the built-in “shrink-wrap” function. In addition, we prepared a spherical applicator space by considering the size of the tumor to be removed from the site where the IORT applicator would be inserted. It was also formed in four parts so that pieces of the EBT3 film could be placed horizontally and vertically between parts.

Based on this new surface, the 3D-printable customized chest phantom was fabricated using a fused deposition-modeling (FDM) 3D printer (DP200; 3D WOX, Sindoh, South Korea) that employed a polylactic acid (PLA) filament with a physical density (ρ) of 1.25 g/cm^3^. The printing parameters comprised a speed of 20 mm/s and layer thickness of 0.2 mm. The other parameters were determined after the calibration. The infill ratios of FDM were determined by the calculated mean HU values of the soft tissue and the lung using the correlation curve between the HU and the infill ratio.

**Figure 2:**
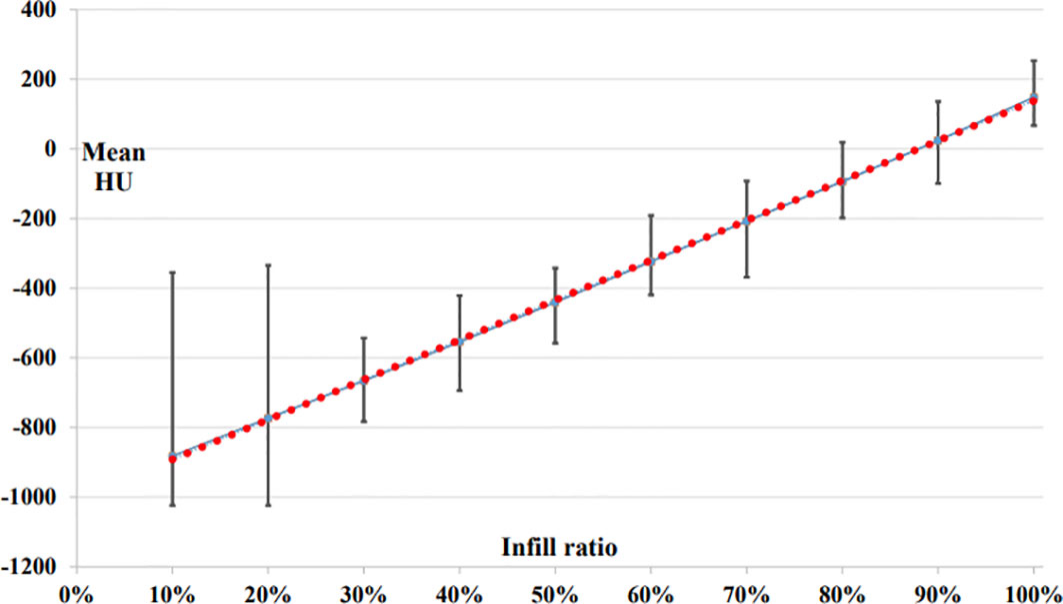
Correlation curve between mean HU and infill ratio.

To create the correlation curve, 10 rectangular samples (width × length × height = 40 mm × 40 mm × 20 mm) were created by varying infill ratios from 10% to 100%. The infill ratios ranged from 0% to 100%, which is the ratio of the volume of a printed thermoplastic to that of air. The samples were printed in a grid pattern using PLA material and the FDM method, which were fabricated by varying the infill ratio by 10%. In total 10 samples, with infill values ranging from 10% to 100%, were scanned to a thickness of 1 mm using a Siemens SOMATOM Definition AS CT Scanner (Siemens Healthcare, Erlangen, Germany), at a voltage of 120 kV and current of 10 mA. All HU measurements, such as the maximum, minimum, and mean, were obtained using an ROI of 30 mm × 34 mm × 15 mm on the scanned CT image. **Figure 2** shows the linear relationship between the infill value (%) and HU as the infill ratios of the 3D printer were increased from 10% to 100% in increments of 10 percentage points. For the linear trend line, we derived an equation of y = 11.438x – 1,005.9, where the R^2^ value was 0.997. As a result, the HU was obtained by varying the infill ratios from 10% to 100%, thereby changing the average HU from −882 to 148.

**Figure 3:**
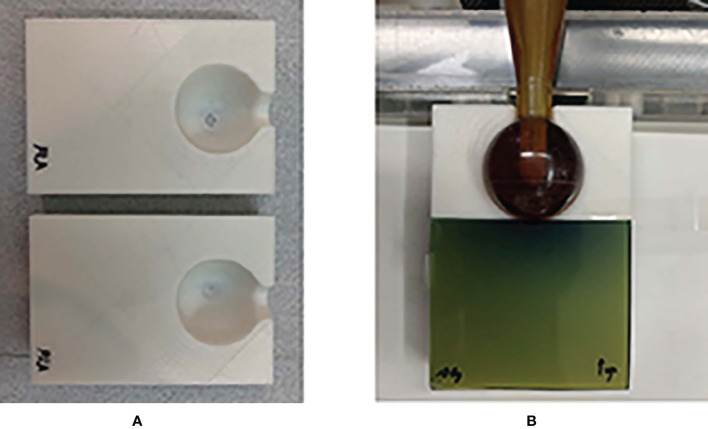
3D-printed water-equivalent phantom made of PLA material. **(A)** The upper and lower parts for the ease of the reproducible placement of a piece of EBT3. **(B)** The film was along the vertical direction relative to the surface of applicator.

The FDM lung model and the soft tissue model were fabricated by applying infill ratios corresponding to the calculated mean HU values. We then assembled the two models into one. The 3D-printed water-equivalent phantom was also fabricated to reflect an infill ratio corresponding to 0 HU to verify the vendor-provided depth dose curve. As shown in **Figure 3**, the water-equivalent phantom was sectioned into upper and lower parts, including a space for the spherical applicator. A piece of EBT3 film was placed between separate parts perpendicular to the spherical applicator.

### Gafchromic EBT3 Film Calibration

A Gafchromic EBT3 film was used for dose measurement by using patient-specific 3D chest phantoms. The EBT3 film consisted of a 28-μm-thick active layer and 125-μm-thick protective layers covering the active layer. Scanning the film before and after irradiation and measuring its optical density allowed us to measure the dose delivered to the film. The optical density of the exposed film was converted into a dose *via* the dose calibration curve.

**Figure 4:**
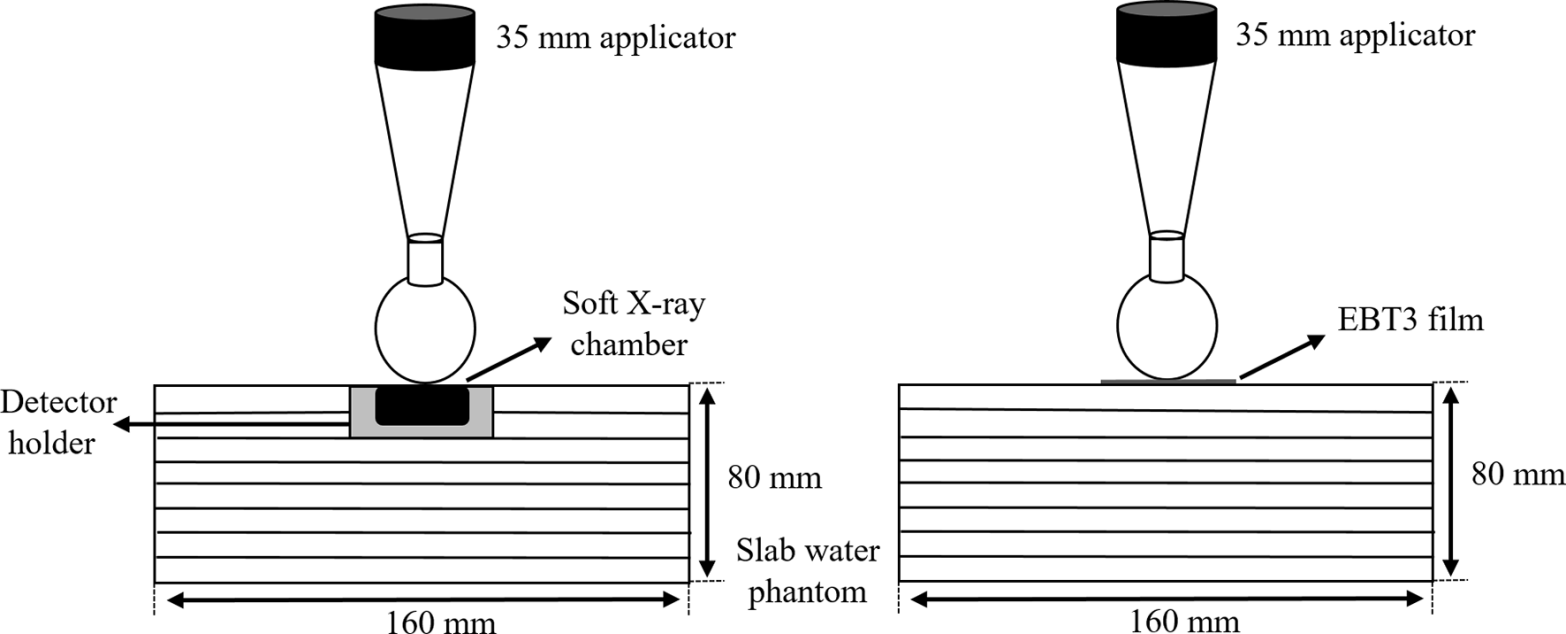
Slab water phantom setup with a parallel plate ionization chamber (left) and an EBT3 film (right) for film calibration.

Dose calibration was performed using a parallel-plate ionization chamber (PTW 23342, Germany) and the EBT3 film on the same INTRABEAM™ system through a conventional technique (20), with exposures ranging from 0 to 20 Gy. The ionization chamber was positioned on the surface of the EasyCube^®^ phantom (Sun Nuclear Corporation, FL, USA), which consisted of water-equivalent slabs of different dimensions and a customized housing holder. The dimensions of the phantom were 16 cm × 16 cm × 8 cm, as shown in **Figure 4**. The absolute dose outputs within the range of doses of interest were measured to determine the durations of treatment in terms of absorbed doses on the phantom surface in contact with the spherical applicator (with a diameter of 35 mm). Each piece of the EBT3 film placed on the surface of the phantom was exposed to nine absorbed doses (0, 0.2, 0.5, 1, 2, 5, 10, 15, and 20 Gy) to cover the full dynamic range of the film. All measurements of the film were performed twice to verify the reproducibility of the results.

**Figure 5:**
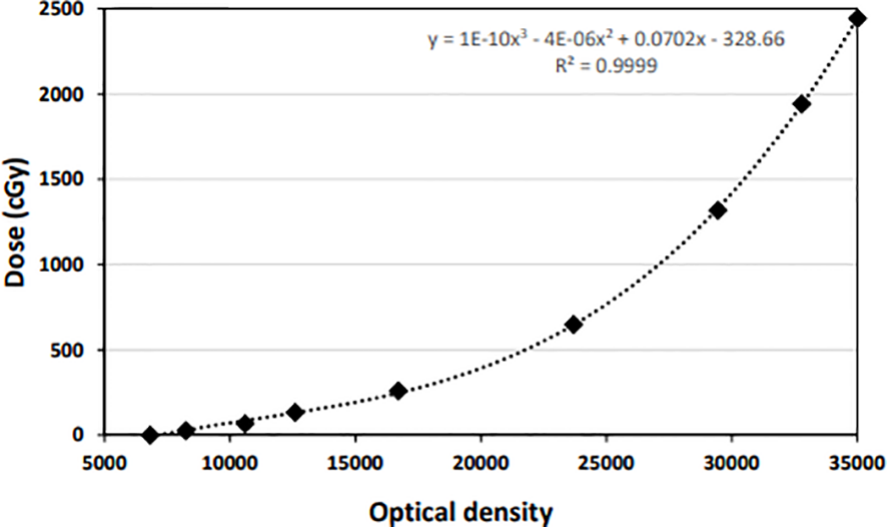
Dose calibration curve for the EBT3 film.

The exposed EBT3 films were digitized using the commercially available Vidar Dosimetry Pro Advantage Red digitizer. The results were analyzed using the RIT version 6.1 software package (RIT, Denver, CO, USA). Uniform ROIs corresponding to the center of each film (10×10 mm^2^ subsets) were chosen for 16-bit red channel calibration. A third-degree polynomial function was used to fit the calibration curve of the film, as shown in **Figure 5**.

To obtain precise and reproducible film dosimetry results, the films were scanned over the same period (24 h irradiation-to-scanning time) after irradiation had been performed at the same orientation because process consistency is crucial to reducing potential uncertainties.

### Measurements and Data Analysis

The Institutional Review Board of the Gangnam Severance Hospital, Korea (IRB No. 3-2017-0033), approved this prospective study in accordance with ethical guidelines and the Declaration of Helsinki.

All measurements were carried out in the 3D-printed water-equivalent phantom as well as the patient-specific 3D chest phantoms created based on five patients receiving IORT. In the water-equivalent phantom, a piece of EBT3 film was exposed parallel to the axis of the beam by placing it along the border of the space for the spherical applicator and between the upper and lower parts of the phantom. This made it possible to bring one side of the film in direct contact with the applicator. The depth dose measured using the film was compared with the depth dose curve provided by the supplier.

**Figure 6:**
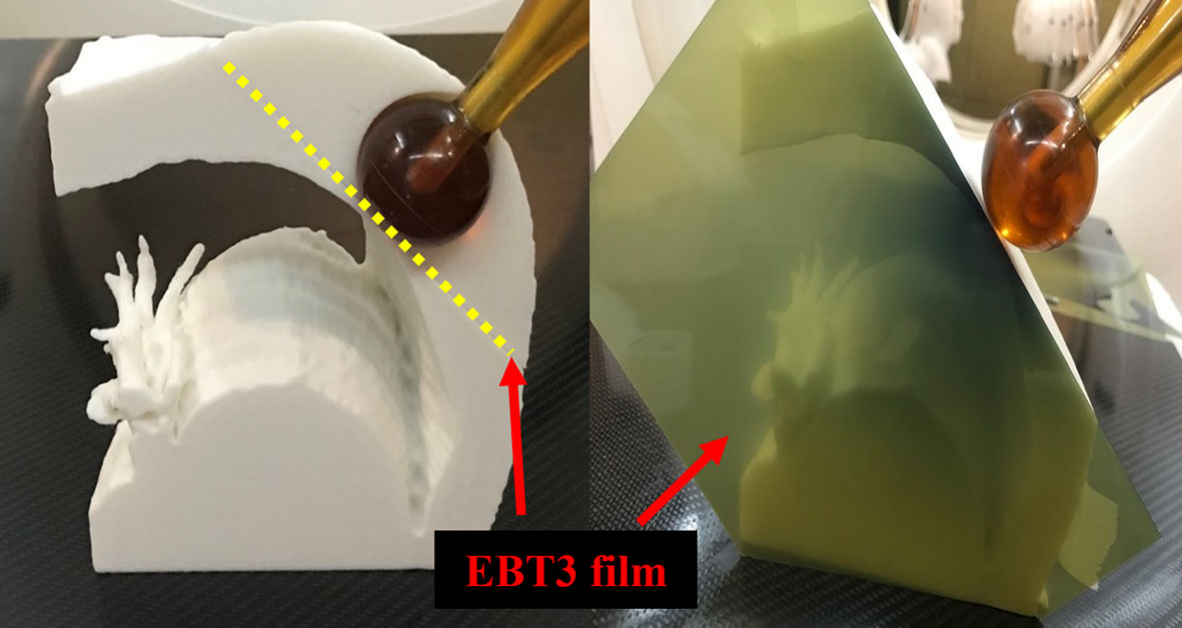
Dose measurement using EBT3 film and patient-specific chest phantom. The spherical application surface dose was measured by placing the EBT3 film on a horizontal plane perpendicular to the axis of the radiation beam (Left), and EBT3 film was placed on a vertical plane parallel to the axis of the radiation beam to measure the depth dose away from the applicator surface (Right).

For the patient-specific 3D chest phantoms, the piece of EBT3 film was placed on a horizontal plane perpendicular to the axis of the radiation beam. The central region of the piece of film was in direct contact with the surface of the spherical applicator, which had a diameter of 35 mm. The other piece of the film was inserted between separate parts of the phantom in a vertical plane parallel to the axis of the radiation beam (**Figure 6**). The treatment duration was calculated such that a 10 Gy dose was delivered on the surface of the applicator.

All exposed films were processed to quantify the dose distributions *via* the dose calibration curve using RIT. A 5 × 5 median filter was applied to each film, and random outliers were excluded from the analysis. The point dose measured from the film placed on the horizontal plane was determined *via* the maximum value extracted from the dose histogram of each irradiated film. Depth dose curves were acquired at depths from 0 to 30 mm through the profile of a piece of film placed on the vertical plane.

## Results

### Evaluations With a 3D-Printed Water-Equivalent Phantom

For precise dosimetric examination, it is necessary to ensure that the 3D-printed density matches the preset density of the infill (14). A CT scan of the 3D-printed water-equivalent phantom was performed to calculate the actual density by comparing it with values in an image value-to-density table. The 3D-printed water-equivalent phantom exhibited a mean of 7 ± 59 HU, in the range from −190 HU to 195 HU. The actual average density was 1.003 g/cm^3^. **Table 1** shows the differences in dose at each depth, between data provided by the vendor and those obtained from the EBT3 film parallel to the axis of the beam, obtained using the 3D-printed water-equivalent phantom. The average of three measurements was reported as the representative estimate, and its estimated uncertainty was based on the absolute value of the difference between each measurement and the average value. The average difference between the vendor and EBT3 film data was 18.3 ± 24.9 cGy (1.8% ± 2.5%), with a difference of up to 64.3 cGy (6.4%) found at a depth of 5 mm. The mean uncertainty in the EBT3 measurement was 16.8 cGy (a maximum of 83.9 cGy at a depth of 0 mm). The measured dose was underestimated at a depth of 0 mm, which corresponded to the border of the piece of film; specifically, this underestimation occurred because of increased uncertainty due to the damage caused by cutting the EBT3 film into pieces. As the depth increased beyond 5 mm, the uncertainty in the measurements performed using the EBT3 film decreased, and a good agreement in terms of absolute dose was obtained between the EBT3 film and the vendor data.

**Table 1:** Comparison between the measured dose from the EBT3 film parallel to the beam axis and vendor-provided depth dose at different depths using the 3D-printed water-equivalent phantom.

Depth [mm]	Measured dose (cGy)				Vender-provided depth dose (cGy)	Dose difference (cGy)	Percent error (%)
	1^st^ trial	2^nd^ trial	3^rd^ trial	Mean ± stdev.			
0	937.2	939.9	1,083.8	987.0 ± 83.9	1,000	−13.0	−1.3
5	493.8	514.9	528.3	512.3 ± 17.4	448	64.3	6.4
10	271.3	281.9	280.9	278.0 ± 5.8	242	36.0	3.6
15	158.9	154.1	161.3	158.1 ± 3.6	145	13.1	1.3
20	100.8	95.6	98.4	98.2 ± 2.6	93	5.2	0.5
25	74.9	70.8	73.1	72.9 ± 2.1	63	9.9	1.0
30	58.5	54.5	56.9	56.6 ± 2.0	44	12.6	1.3

Percent error (%) = dose difference/prescription dose × 100%.

### Evaluations With 3D-Printed Patient-Specific Chest Phantoms

**Figure 7:**
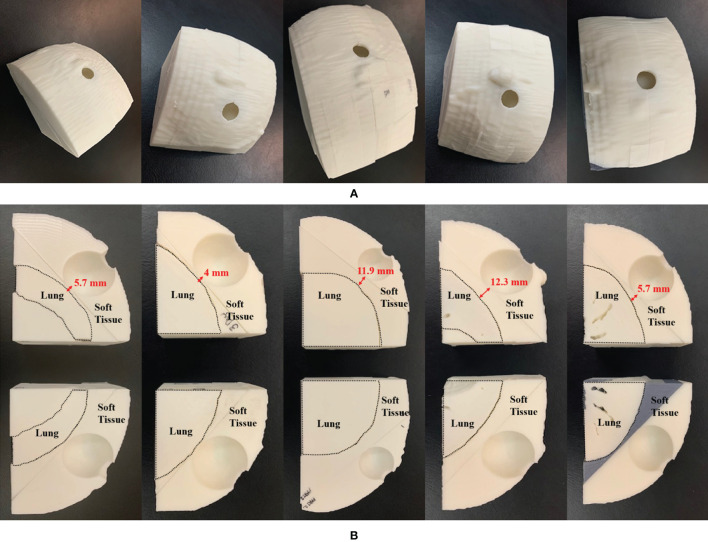
3D-printed patient-specific chest phantom: **(A)** A set of phantoms representing the soft tissue and lung, and **(B)** cross-sections of the soft tissue and lung of the five patients. The left-to-right columns are from PSQA1 to PSQA5.

**Figure 8:**
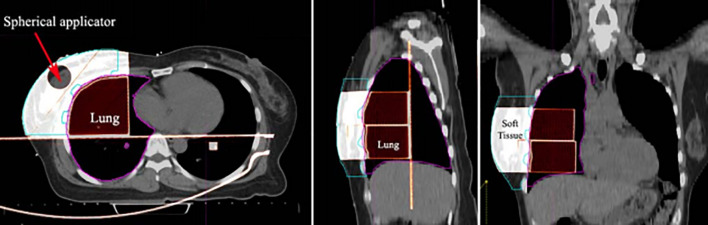
Image fusion between the patient’s CT and the 3D-printed phantom CT.

Chest phantoms of five patients were fabricated for PSQA measurements. IORT patients were randomly selected and the applicator was used for PSQA measurements. **Table 2** shows the location of the dose measurement for each patient and the distance between the tumor and the lung. The mean and standard deviation of the HU values of the voxels inside each ROI (soft tissue and lung) were calculated. Based on an infill ratio corresponding to the mean HU value, the customized chest phantoms were fabricated using the 3D printer, as shown in **Figure 7**. The 3D-printed soft tissue and lung were sectioned in four and two parts, respectively, to facilitate the reproducible positioning of the spherical applicator and pieces of film. **Table 3** shows the HU values of the soft tissue and the lung parts for the five patient-specific 3D chest phantoms. On average, differences in the HU between the patient and the phantom were 2.2 HU for soft tissue and −26.2 HU for the lung. This was in close agreement with the expected HU in terms of the calculated doses. Because each part was printed sequentially, it took 19~50 h to fabricate a single phantom depending on the amount of filament used. The weight of the filament used ranged from 329 g to 975 g. The 3D printing process required only approximately 30 min of labor to fill, clean, assemble, and verify the printed phantom. The infill ratio of printing ranged from 83% to 85% for soft tissue and 18% to 37% for the lung. The Dice similarity coefficient (DSC) was considered to quantify the similarity between the corresponding structures. The DSC ranges from 0 (no overlap) to 1 (perfect overlap). A large DSC value indicates good overlap between the 3D-printed phantom and the patient. As shown in **Figure 8**, CT images of the patients and those of their corresponding phantoms were visually matched and then fused to calculate the DSC in the soft tissue and the lung, respectively. On average, the DSC between the patient and the phantom was 0.97 for the soft tissue and 0.97 for the lung.

**Table 2:** Information on the five patients who received breast IORT.

Patient no.	Site	Distance between the tumor and the lung	HU of patient CT (mean ± st. dev.)	
			Soft tissue	Lung
Patient 1	Left upper	≤1 cm	−60 ± 53 HU	−582 ± 182 HU
Patient 2	Left upper	≤1 cm	−38 ± 52 HU	−800 ± 158 HU
Patient 3	Right upper	>1 cm	−44 ± 55 HU	−725 ± 140 HU
Patient 4	Left lower	>1 cm	−61 ± 53 HU	−803 ± 153 HU
Patient 5	Right lower	≤1 cm	−37 ± 59 HU	−713 ± 162 HU

**Table 3:** Printing information, HU, HU difference, and Dice similarity coefficient (DSC) for the five patient-specific 3D chest phantoms fabricated by using a 3D printer.

	PSQA 1	PSQA 2	PSQA 3	PSQA 4	PSQA 5
Printing time (soft tissue + lung)	2,994 min.	1,147 min.	3,049 min.	1,441 min.	1,731 min.
Amount of filament	615 g	329 g	975 g	404 g	452 g
HU of phantom CT (mean ± st. dev.)					
Soft tissue	−48 ± 29 HU	−42 ± 29 HU	−49 ± 92 HU	−56 ± 74 HU	−56 ± 83 HU
Lung	−580 ± 96 HU	−793 ± 154 HU	−680 ± 124 HU	−762 ± 184 HU	−705 ± 172 HU
HU Difference between patient CT and phantom CT					
Soft tissue	−12	4	5	−5	19
Lung	−2	−7	−45	−41	−8
DSC between patient CT and phantom CT					
Soft tissue	0.97	0.97	0.97	0.98	0.97
Lung	0.97	0.98	0.97	0.96	0.97

**Table 4** shows the differences between the 1,000 cGy dose administered on the surface of the 35-mm spherical applicator and the dose measured from the pieces of film perpendicular to the axis of the beam. The film measurements ranged from 930.2 cGy to 1,025.6 cGy for the five patient-specific chest phantoms. An evaluation of these measurements versus the prescribed dose indicates that the mean dose difference was −21.6 ± 39.1 cGy, and the mean percentage error was −2.16% ± 3.91%.

**Table 4:** Comparison between the measured dose from the EBT3 film placed on the horizontal plane perpendicular to the beam axis and the prescription dose using five patient-specific chest phantoms.

No.	Prescription dose (cGy)	Measured dose (cGy)	Measured dose – prescription dose (cGy)	Percent error (%)
PSQA 1	1,000	992.2	−7.8	−0.78
PSQA 2	1,000	930.2	−69.8	−6.98
PSQA 3	1,000	997.5	−2.5	−0.25
PSQA 4	1,000	946.5	−53.5	−5.35
PSQA 5	1,000	1025.6	25.6	2.56

**Figure 9:**
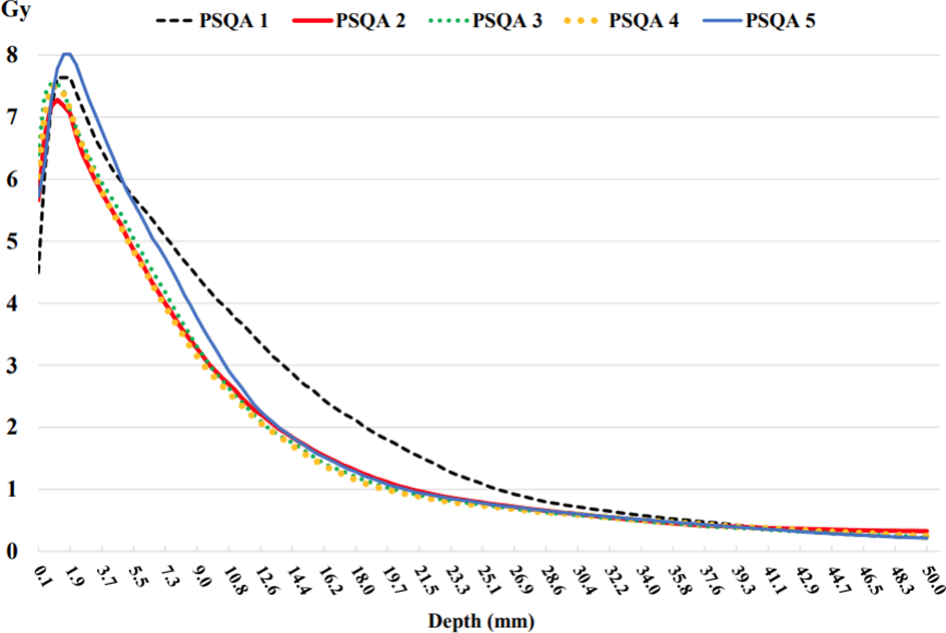
Depth doses measured from the EBT3 film parallel to the beam axis using the five 3D chest phantoms on the INTRABEAM™ system using a 35 mm spherical applicator.

**Figure 9** shows the depth dose measurements of the five PSQA phantoms using pieces of film parallel to the axis of the beam. The measured dose at shallow depths along the border of the film had been underestimated. A depth of 2 to 3 mm was found to be adequate to initiate the depth dose curve. This implies that the first few millimeters along the border of the film could not be used when performing parallel film measurements because the EBT3 film was cut into pieces. This finding is consistent with those of previous studies (21, 22), which have reported damage to the film pieces. Unlike megavoltage-scale X-rays, kilovoltage-scale depth dose curves produced by INTRABEAM™ led to a steep fall-off in the dosage with depth. This is beneficial to the organs at risk in the environment of the lesion and allows radiation oncologists to prescribe higher doses to the lesion itself.

## Discussion

Few studies have examined PSQA for IORT. This subject is of particular concern in the context of single-session radiotherapy techniques such as IORT where there is no opportunity to compensate for possible errors in later treatment sessions. In light of this, this study developed a simple and non-toxic PSQA method for IORT using 3D printing. The results confirm the feasibility of PSQA for IORT using a 3D printer and a conventionally used PLA filament to fabricate a patient-specific chest phantom. FDM printers are the most economical 3D printers, and they will be advantageous for future clinical applications. In this study, only one FDM-type 3D printer was used to fabricate the patient-specific chest phantoms. The fabricated phantoms can be evaluated in terms of the lung (−803 to −582 HU) and soft tissue (−61 to −37 HU), which have a lower physical density than water, by changing the infill ratio. Although differences in HU values between the patients and the phantoms were observed (the ΔHU of soft tissue = −12–19 HU and ΔHU of lung = −45–2 HU) in our experimental environment, the dosimetric influences of these differences were not significant, based on the fact that it is appropriate to set tolerances of ±20 HU for soft tissue and ±50 HU for lungs when restricting changes in dose in the treatment plan to within ±1% (23).

For dose measurements using the patient-specific chest phantom assembled in the manner described above, the films were exposed along two directions: perpendicular and parallel to the axis of the beam. On the surface of the applicator, the horizontal plane was better than the vertical plane because the border of the piece of film could be damaged. In a depth dose comparison using a 3D-printed water phantom, the average difference between the measurements provided by the vendor and those of the actual EBT3 film was the greatest at a depth of 5 mm, but a substantial difference in the dose gradient was observed between 0 and 10 mm; this is because the dose decreased by 75% from the surface of the applicator to a depth of 10 mm. In this case, the main cause of uncertainty in the measurement was the positioning of the detector. As has been determined in previous studies (24, 25), both the ionization chamber and the dosimetry of the film can be performed with an accuracy of 5%–10%, when considering the largest error due to the positioning of the dosimeter. Based on this fact, the steep dose gradients produced by the INTRABEAM system yielded a 10% difference in dose for a 1 mm difference in distance. This inherent variation, combined with the uncertainties involved in practical measurements using different dosimeters, may preclude even a 5% tolerance in the depth doses. Therefore, a 10% dose difference may be acceptable at a steep dose gradient.

The morphology of patients was accurately reproduced to fabricate customized phantoms. The designs produced here were found to be robust and can be easily modified to add strength as needed. If 3D-printed parts break, they can be accurately reproduced with minimal additional labor. With more time, it will be possible to produce 3D-printed phantoms with even greater heterogeneity. It is evident that this has copious potential benefits for the stage of clinical adaptation. Therefore, this 3D printing technology can be employed to fabricate accurate shapes that reflect the spherical applicator and patients’ anatomical structure, which in turn makes it possible to simultaneously review the absolute dose to the lesion and the surrounding organs at risk. This improves patient safety through the PSQA prior to IORT. The digital workflow ensures the accuracy and reproducibility of the procedure.

This study on prototype phantoms for IORT-specific PSQA involves a few practical considerations. First, our study focused on structures with low densities, by excluding bone structures, because the surface of the spherical applicator was attached to the soft tissue between the ribs. To incorporate the bone structure by using 3D printing, we can assemble the structure using an FDM lung and soft tissue model to replicate the CT values of the bones using color-jet printing materials with more than 1,000 HUs (26). For further investigation, contrast agents with different concentrations can be injected into voids inside the 3D-printed part to obtain a higher range of up to +1,000 HU (27). Second, the experiment used only five cases, as printing individual phantoms is time and resource intensive. On average, a single phantom fabrication took 35 h. This 3D printing process might not be practical for general PSQA. However, given the rapid pace of development of 3D printing at present, these technologies are expected to improve dramatically over the next few years to provide better convenience, speed, and precision. Third, standard printing procedures should be used to ensure consistency. Special caution should be taken regarding the first layer of each print, as distortion can be caused by the material not completely sticking to the print bed.

Our study has some limitations. First, the PSQA phantom fabrication described in this study may not adequately reproduce all the uncertainties arising in clinical settings. In the breast IORT procedure, a spherical applicator is placed inside the tumor cavity, then the wall of the tumor cavity is pulled firmly to the applicator surface using a purse-string suture. This process can lead to soft tissue compression due to pre-fixation pressure as well as potential air gaps near the applicator surface in some areas of the breast. Additionally, contrary to our study assuming that the position of the applicator in the intercostal space excludes bone-equivalent heterogeneity in the phantom, in some cases during this fixation process the position of the applicator may be on the ribs. In this case, there may be a significant dose in the ribs. Second, skin and subcutaneous tissue doses are a significant problem with breast IORT. In our study, skin measurements were not taken into account in the PSQA phantom because an optically stimulated luminescence dosimeter was clinically used for in vivo dosimetry to detect radiation doses delivered to the skin during breast IORT (28). However, the most common late toxicity, with a maximum appearance four years following treatment, is telangiectasia. The development of telangiectasia is strongly correlated with the doses applied to the subcutaneous vessels. Measuring the skin dose on the PSQA phantom can help prevent the onset of severe telangiectasia after several years. Therefore, PSQA phantom development must incorporate this skin measurement prior to patient treatment; this leads to a more secure treatment through personalized pretreatment dosimetry investigations. Nevertheless, owing to the absence of sufficient QA data regarding IORT, our method for creating a simplified phantom composed of soft tissue and lung models could be an important milestone in mimicking personalized dosimetry.

## Conclusions

In this study, patient-specific 3D-printed chest phantoms were successfully constructed to simulate the IORT-related dose distributions to cancerous tumors, surgical tumor bed, and the surrounding low-density organs such as lungs and soft tissue. This allows not only for the prediction of the depth dose at various distances from the spherical applicator, but also for the verification of the dose by comparing the prescribed dose to the expected dose. The proposed 3D printing methodology provides a viable and inexpensive method for fabricating variable-density solid phantoms for breast IORT-specific PSQA. This enhanced precision offers new opportunities for advancing IORT.

## Data Availability Statement

The original contributions presented in the study are included in the article/supplementary material. Further inquiries can be directed to the corresponding author.

## Ethics Statement

The studies involving human participants were reviewed and approved by The Institutional Review Board of the Gangnam Severance Hospital, Korea (IRB No. 3-2017-0033), in accordance with ethical guidelines and the Declaration of Helsinki. The patients/participants provided their written informed consent to participate in this study. Written informed consent was obtained from the individual(s) for the publication of any potentially identifiable images or data included in this article.

## Author Contributions

The data collection, conception, design, and drafting of the manuscript were performed by YHC and HL. The data analysis and interpretation were performed by HL, KP, and IJL. The final manuscript was edited by IJL. Patient enrollment were performed by YAC, JWK, KRP, and IJL, who also contributed to useful discussions on the manuscript. All authors contributed to the article and approved the submitted version.

## Funding

This study was supported by the Basic Science Research Program through the National Research Foundation of Korea, funded by the Ministry of Education (2019R1I1A1A01062157), and by a grant of the Korea Health Technology R&D Project through the Korea Health Industry Development Institute (KHIDI), funded by the Ministry of Health & Welfare, Republic of Korea (HI19C1330).

## Conflict of Interest

The authors declare that the research was conducted in the absence of any commercial or financial relationships that could be construed as a potential conflict of interest.
